# Project SEARCH (Scanning EARs for Child Health): validating an ear biometric tool for patient identification in Zambia

**DOI:** 10.12688/gatesopenres.13197.1

**Published:** 2020-11-06

**Authors:** Lauren Etter, Alinani Simukanga, Wenda Qin, Rachel Pieciak, Lawrence Mwananyanda, Margrit Betke, Jackson Phiri, Caroline Carbo, Arnold Hamapa, Chris Gill

**Affiliations:** 1 Department of Global Health, School of Public Health, Boston University Medical Campus, Boston, MA, 02118, USA; 2 College of Engineering, Boston University, Boston, MA, 02215, USA; 3 Department of Computer Science, School of Natural Sciences, University of Zambia, Lusaka, Zambia; 4 Department of Computer Science, College of Arts and Sciences, Boston University, Boston, MA, 02215, USA; 5 Right to Care Zambia, Lusaka, Zambia

**Keywords:** biometrics, patient identification, electronic medical records, global health

## Abstract

Patient identification in low- to middle-income countries is one of the most pressing public health challenges of our day. Given the ubiquity of mobile phones, their use for health-care coupled with a biometric identification method, present a unique opportunity to address this challenge. Our research proposes an Android-based solution of an ear biometric tool for reliable identification. Unlike many popular biometric approaches (e.g., fingerprints, irises, facial recognition), ears are noninvasive and easily accessible on individuals across a lifespan. Our ear biometric tool uses a combination of hardware and software to identify a person using an image of their ear. The hardware supports an image capturing process that reduces undesired variability. The software uses a pattern recognition algorithm to transform an image of the ear into a unique identifier. We created three cross-sectional datasets of ear images, each increasing in complexity, with the final dataset representing our target use-case population of Zambian infants (N=224, aged 6days-6months). Using these datasets, we conducted a series of validation experiments, which informed iterative improvements to the system. Results of the improved system, which yielded high recognition rates across the three datasets, demonstrate the feasibility of an Android ear biometric tool as a solution to the persisting patient identification challenge.

## Introduction

One of the most pervasive public health challenges in low- and middle-income countries (LMICs) is the provision of comprehensive and coordinated longitudinal healthcare ^1^. The success of disease management programs and primary care hinges upon the ability to accurately identify patients repeatedly, when and where they seek care. The inability to identify individuals across time and space drastically compromises the effectiveness of public health programs and interventions to deliver the right care to the right people at the right time ^2^.

There are many challenges to patient identification in LMICs including the limited availability or sheer absence of national insurance programs, the difficulty of disambiguating common names (a problem compounded by high rates of illiteracy and the lack of standardized spellings), and the absence or unreliability of birth records ^3– 5^. Absent a robust solution to the patient identification problem, the promise of centralized medical records cannot be realized, and the goal of providing quality longitudinal care will remain elusive ^6^.

To address this problem, we launched Project SEARCH (Scanning EARs for Child Health) in 2014 with the goal of developing a mobile health (mHealth) solution for individual identification using biometric analysis of ear morphology ^7^. Early work in the project focused on proof of concept, identifying the best pattern recognition algorithm (PRA) and standardizing the image capture process ^8, 9^. By definition, biometric data cannot be lost, left at home, sold, or traded, offering distinct advantages over external identifiers. Biometric analysis of ears has many advantages over fingerprinting or iris scanning ^10^. Fingerprints require external scanners which can be expensive, and acceptability is a barrier due to the association between fingerprints and law or immigration enforcement. Moreover, fingerprint scanners struggle to identify infants whose finger whorls are too shallow for detection ^11^. Similarly, iris scanning requires external sensors, and that a user follow instructions, therefore often failing in infant populations. By contrast, ears are anatomically unique, easily accessible, and impersonal ^12, 13^. Additionally, ears can be sampled using a phone camera, without the need for external sensors ^14^.

The SEARCH system combines hardware and software solutions for optimizing ear identification and verification.

**Figure 1.**
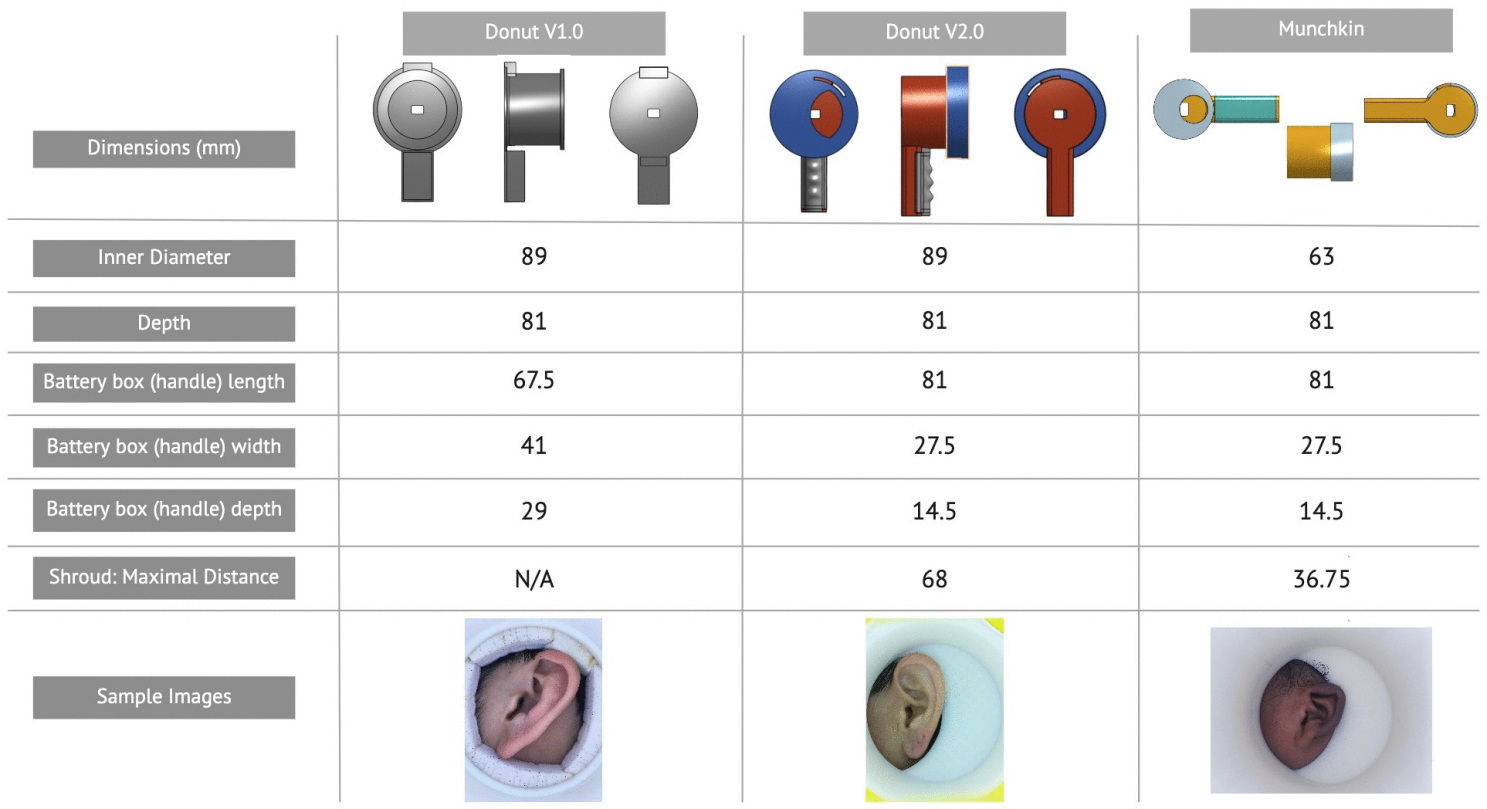
Dimensions for each version of the Donut/Munchkin. The Figure summarizes the sequential design innovations in the design of the Donut. v1 was the first to include internal lighting as an attempt to standardize illumination. Further refinements included the use of a voltage regulator to control dimming as batteries drain down. v2 included a shroud. This is a semi-circular opening in an otherwise opaque plastic shield at the mouth of the Donut. The goal is to isolate the ear from surrounding distracting anatomy, such as the neck line but particularly hair. The purpose is to reduce extraneous information being presented to the SIFT algorithm. v3 is a Donut sized for very small babies, and so was dubbed ‘the Munchkin’ given our allegiance to the city of Boston and our enjoyment of caffeine and sweets. The Munchkin’s diameter is much smaller. The adult sized Donut’s aperture was so large that the entire head of small babies could fit in in some cases, and in all cases it was difficult to center the device when trying to isolate the ear. Another change is that the handle of the Munchkin, which in versions of the Donut is used to mount the smart phone, is rotated 90 degrees relative to v1 and v2. This is also a response to the small size of infants, whose shoulders got in the way of the v1 v2 handles, making it hard to take the picture. By rotating the handle forward towards the infants’ noses, the anatomic interference is removed.

The hardware, termed “the Donut”, is a light-opaque cylinder (with a case for mounting a smartphone) that reduces sources of error during image capture by: 1) standardizing the distance between the side of the head and the camera, 2) minimizing motion and variation in approach (yaw, pitch, and roll), and 3) standardizing lighting intensity by providing its own illumination using internal 360 degree LED lighting strips powered off a 9V battery ( Figure 1). Essentially, the Donut is a device that allows one to take high quality, reproducible images of ears. As previously published, the Donut was found to be an essential component to the success of the SEARCH system, improving top-1 matching accuracy from 24% to 96.5% ^15^.

On the software side, the SEARCH system uses a simple, yet robust pattern recognition algorithm known as Scale Invariant Feature Transform (SIFT) ^16^. This algorithm transforms the picture of the ear into a set of descriptors corresponding to regions of interest on the image. These descriptors are compared across images creating a series of vector diagrams. Based on the average Euclidean distance between descriptors, where smaller distances correspond to stronger matches and larger distances weaker matches, a list of top ranked matches is determined ( *Methods*).

Results from this early work gave us confidence that the SEARCH system was viable ^17, 18^, leading to the current NIH supported project. In this paper, we describe the incremental and iterative process of validating our system.

Our goal was to optimize the performance of the system by presenting it with ear image datasets from three cohorts of increasing complexity, with the final cohort being the target user group for the mature system, namely infants in Zambia. We made iterative improvements to the system through a combination of image processing and database filtering techniques to address these challenges. The goal was a highly robust system for biometric subject identification that is simple, non-invasive, acceptable, and highly accurate.

## Results

**Figure 2.**
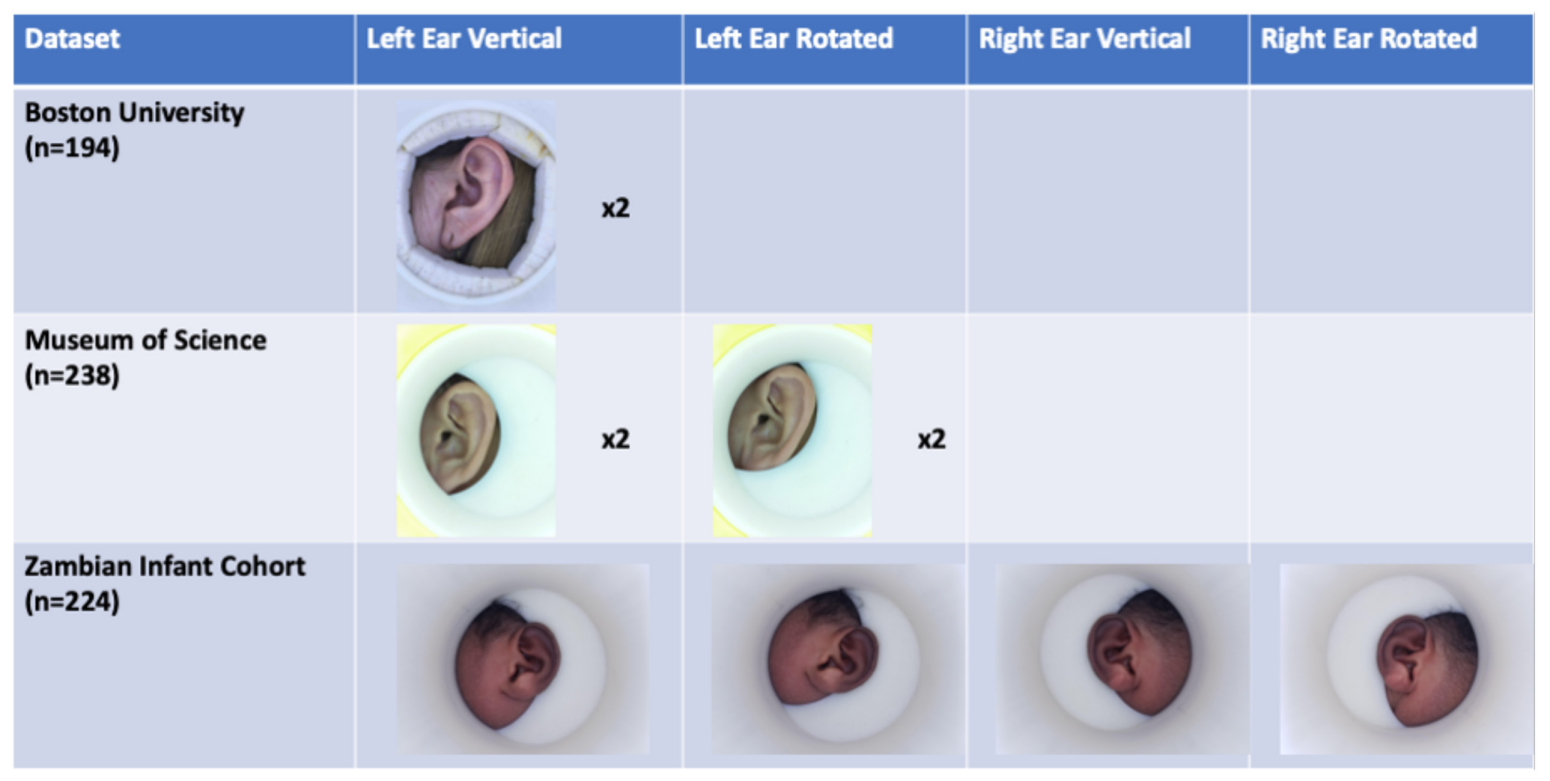
Representative samples from the three datasets. Cohort 1 (Boston University) used Donut v1,a light-opaque cylinder with an LED lighting strip inlaid along the inner circumference, powered by a 9V battery, and complete with a bubble level to control for angle rotation. Cohort 2 (Boston Museum of Science) used Donut v2, which incorporated the same lighting, and an internal, rotating shroud to cup the ear, thereby removing random noise from images due to hair, neckline, or other features that might distract the algorithm. Rotated images were introduced in Cohort 2. By taking two images that are slightly offset, and combining those images through the process of concatenation, fortuitous SIFT points that emerge only from particular angles relative to the light source tend to drop out. This leaves a higher proportion of constant SIFT points for analysis, thereby optimizing the signal to noise ratio. Cohort 3 (Zambian Infant Cohort) used Donut v3, a smaller diameter cylinder dubbed ‘the Munchkin’, which was designed for use in very small infants. It also includes a deeper shroud to better center the image and further mask extraneous features from the image. Type or paste legend here. Paste figure above the legend.

Over the course of the project, we collected a total of 2,244 ear-images from 658 individuals to create three datasets of ear images (see representative examples, Figure 2). The first, consisting mainly of Boston University undergraduates (Cohort 1, N=194), was the most homogeneous and least challenging from the perspective of subject identification. The second cohort was comprised of attendees at the Boston Museum of Science (MOS) (Cohort 2, N=238), and represented a more heterogeneous population, captured over a longer period (12 months) by six different data collectors from 2018–19, thereby introducing variation from inter-user technique. The third cohort was our target end-user population, Zambian infants. This was collected among newborns and young infants attending the Chawama Clinic health center in Zambia’s capital city of Lusaka by a single data collector in the fall of 2019 (Cohort 3, N =224).

Each dataset represents a specific point and time in the project. The iterative approach taken to optimize our SEARCH system (Donut and identification algorithm) is a story best told through the validation of the system across these three datasets. We collected data using different versions of the Donut, minimizing variables present during image capture, then conducted the identification step using SIFT. Our previously published work demonstrates the essentiality of the Donut ^9^. While refinements were made to the Donut, no further experiments were designed to test how these design changes effect performance. The following results focus on software techniques that were added to our algorithm to improve performance.

Table 1 displays the demographic breakdown and data collection strategies for each dataset. Age, sex, and racial composition of each cohort differed markedly. Overall, Cohort 2 had the widest age range of participants, and Cohort 3, unsurprisingly, the least since all were enrolled at either one week or 14 weeks of age. 100% of the Zambian cohort were African, whereas only ~3–4% of cohorts one and two were African American.

**Table 1.** Dataset Characteristics.

	*Boston University* *(n=194)*	*Museum of* * Science (n=238)*	*Zambian Infant* * Cohort (n=224)*
***Sex***			
* Female*	78 (40.2%)	147 (61.8%)	109 (48.7%)
* Male*	116 (59.8%)	91 (38.2%)	115 (51.3%)
***Age (years)***			
* Mean (SD)*	23.6 (8.44)	27.9 (15.5)	0.138 (0.148)
* Median [Min, Max]*	21.9 [19.5,84.8]	24.3 [3.79, 83.6]	0.0219 [0.0110, 0.482]
***Race/Ethnicity***			
* African American*	6 (3.1%)	9 (3.8%)	0 (0%)
* American Indian*	0 (0%)	1 (0.4%)	0 (0%)
* Asian*	45 (23.2%)	22 (9.2%)	0 (0%)
* Caucasian*	120 (61.9%)	161 (67.6%)	0 (0%)
* Hispanic*	13 (6.7%)	23 (9.7%)	0 (0%)
* Other*	10 (5.2%)	22 (9.2%)	0 (0%)
* Zambian*	0 (0%)	0 (0%)	224 (100%)
***Donut Version***			
* Donut Version 1.0*	194 (100%)	0 (0%)	0 (0%)
* Donut Version 2.0*	0 (0%)	238 (100%)	0 (0%)
* Munchkin*	0 (0%)	0 (0%)	224 (100%)

****Experiment one: initializing the SEARCH algorithm, Cohort 1 results****. Our early experiments through Project SEARCH were simulated using the engineering software program, MATLAB, allowing full experimental flexibility as we initially tested and developed the system. However, MATLAB is proprietary software, and not a deployable platform for the final application, which would need to run on Android OS. Between these two extremes, we still needed a computer-based experimental program with high coding flexibility, to test and validate software improvements efficiently prior to adapting them to the far less flexible Android OS platform.

To bridge this gap, we implemented the algorithm on a new experimental platform using Python. We chose Python for its similarity to Java (the programming language used for our Android OS platform) knowing that any changes we made on Python could easily be translated to the Android application.

We measured performance of the Python implementation using our earliest available dataset, Cohort 1. The Boston University cohort provided an easy target for success: recognition accuracies were markedly high, achieving top-1 and top-10 performances of 96.9%, and 98.97% ( Table 2).

**Table 2.** Top-1 and Top-10 identification accuracies of the Boston University dataset (Cohort 1) using our Python platform.

	Top-1 Accuracy	Top-10 Accuracy
**Boston University Dataset** **(Cohort 1, N=194)**	96.9%	98.97%

****Experiment two: improving the accuracy of SEARCH within the Museum of Science Dataset.**** Paradoxically, this early success was problematic given that the Boston University dataset quickly hit a ceiling threshold. We attribute the dataset’s high initial performance to the homogeneity of Cohort 1: the dataset was made up of a racially homogeneous group of Boston University undergraduate students and their professors, most of whom were Caucasian. Moreover, all participants were photographed by one study team member who was, at that point, experienced in taking high quality ear images, thereby minimizing random variation due to photographic technique. In short, the library of ear images from Cohort 1 was too good, and thus not representative of real- world conditions.

To present a greater challenge to the algorithm, we partnered with the Boston Museum of Science (MOS) to create a second cohort from a more heterogeneous population of museum attendees. Cohort 2 was markedly more complex than Cohort 1. Images were taken by a larger group of six data collectors, introducing a greater degree of random variation due to user technique. Moreover, the 238-participant cohort was more racially diverse and included participants across a wide range of ages. In moving from Cohort 1 to Cohort 2, the performance of the SIFT algorithm fell sharply, forcing us to build back the accuracy through further refinements in our system. This tool kit of improvement strategies is grouped into three categories laid out in Table 3: image pre-processing, post-processing, and database management.

**Table 3.** Design innovations toolkit.

Pre-Processing Techniques		
Innovation	Description	Dataset(s)
Manual Crop	*A manual crop is applied to isolate the ear in the photograph. Reduces photo size* *by 2/3rds.*	Cohort 1, Cohort 2, Cohort 3
Resize to smaller pixel count	*Resizing drastically decreases the size of the image. Improves image processing* *time and minimizes white noise in the image. In our case, the width of the ear* *is set to a specific pixel count, and the length of the ear is resized to maintain* *proportions of the original image.*	Cohort 1, Cohort 2, Cohort 3
Histogram Equalization	*An image enhancement technique that improves contrast within an image by* *spreading out the most frequent pixel intensity values. This has the effect of* *sharpening edges, such as the contour of the ear.*	Cohort 2, Cohort 3
Post-processing Techniques		
Innovation	Description	Dataset(s)
Key Points Concatenation	*SIFT descriptors from multiple images of the same ear are combined, creating* *a composite vector. This composite vector is stored as the individual’s ID. With* *concatenation, matching is done by computing distance scores between composite* *vectors. This has the effect of eliminating many false positive descriptor points,* *which might fortuitously occur only in one image, but disappear with subtle* * changes of lighting angle, while retaining constant descriptors. This improves the* *signal to noise ratio.*	Cohort 2, Cohort 3
Database Management Techniques		
Innovation	Description	Dataset(s)
Gender Filter	*Each identifying vector is tagged with a gender. Before searching the database for * *a match, the database is narrowed to include only descriptors that match with the* *gender of the descriptor in question.*	Cohort 2, Cohort 3

Prior to data collection at the museum, we introduced mechanical design refinements to the Donut, adding a rotating shroud which provided two main advantages over the previous design. First, it cupped the ear with a curved shape, blocking out background distraction such as hair, and helping to center ears in the frame. Second, it rotated 30 degrees providing the option to take pictures at two different angles, which allowed for the post-processing step of concatenation.

Referring to the tool kit laid out in Table 3, our first analysis involved applying two pre-processing techniques: a manual crop and resize. These techniques were applied to all images in the MOS dataset, yielding top-1 and top-10 accuracies of 80.25% and 89.07%, respectively. Since resizing appeared to have a strong influence on both the speed by which the algorithm made its matches, and the accuracy of the matches themselves, we tested how our cropped MOS dataset performed at different resizing parameters. Results suggest top-1 performance is optimal when images are resized to a set ear-width of 150 pixels ( Table 4).

**Table 4.** Top-1 and Top-10 identification accuracies of the MOS dataset (Cohort 2, N=238) at different resizing settings using our experimental platform (Python).

Width (pixels)	Top-1 Accuracy	Top-10 Accuracy
**75**	77.73%	88.65%
**100**	77.73%	92.01%
**125**	77.73%	88.65%
**150**	80.25%	89.07%
**175**	73.94%	89.07%
**200**	76.47%	87.81%

Performance as a result of cropping and resizing was still well below what was seen in the BU dataset (top-1, 96.92%, top-10, 98.97%). Therefore, we next report on the effects of incorporating additional techniques into our algorithm (pre-processing, post-processing, and database management).

Over the course of data collection at the MOS a 12-month period, the 9V battery that powered the Donut dimmed considerably, and though it was changed periodically, this still resulted in variable and occasionally poor illumination. While this was unintentional, it reemphasized the strong impact of lighting intensity on matching rates and suggested a further refinement to our system. Therefore, to account for differences in illumination, we applied the image enhancement technique of histogram equalization (HE). In applying HE to our cropped and resized data we improved the top- 1 performance by 6%, and top-10 by 5% ( Table 5). While HE improves our top-1 and top-10 accuracies, we call attention to the order in which techniques are applied. When HE is applied without first applying a manual crop, it darkens the entire image, and results in a considerable decrease in performance ( Table 5). The increased contrast yielded through HE is visually evident, and led to a substantial increase in identification yield vs. cropping/resizing alone, yielding top-10 matching rates exceeding 90% ( Table 5). This further emphasizes the critical need for cropping and resizing and also demonstrated that these techniques could be used in combination to yield improved accuracy.

**Table 5.** Top-1 and Top-10 accuracies associated with applying different processing and database management techniques to our MOS dataset (Cohort 2, N=238) using our Python platform.

	Pre-processing			Post - processing	Database manipulation	Percent matched	
Strategy	Manual Cropping	Resizing to 150 Pixels	Histogram Equalization (HE)	Key Points Concat- enation	Gender Filter	Top-1	Top-10
**One**		**X**				76.9%	91.6%
**Two**		**X**	**X**			50.0%	76.1%
**Three**	**X**	**X**				80.3%	89.1%
**Four**	**X**	**X**	**X**			87.0%	94.1%
**Five**	**X**	**X**	**X**	**X**		97.1%	98.7%
**Six**	**X**	**X**	**X**	**X**	**X**	100.0%	100.0%

Performance was further enhanced through the sequential addition of key points concatenation, a technique for improving the signal to noise ratio of the images, and a gender filter, a data-base manipulation that reduces the risk of false positives by narrowing the size of the data set being queried to individuals of the same sex. Through the combination of these techniques, we were ultimately able to achieve 100% matching rates within the top 1 and top 10 most likely matches ( Table 5).

****Experiment three: testing the revised SEARCH algorithm in the end-user population of Zambian infants.**** One major limitation up to this point is that the datasets collected are not representative of our intended use-case population: Zambian infants. As a logical progression, we anticipated that this cohort would be the most challenging of all, being collected in a real-world clinic setting. Moreover, the population was very different, being entirely comprised of very young African infants. The small size of the infant ears mandated the development of a new, smaller Donut for image capture, which we dubbed “the Munchkin” ( Figure 1). This was necessary when it proved that the larger Donut was too large relative to smaller infant head sizes, and could not easily make close contact for image capture. It also includes a deeper shroud to better center the image and further mask extraneous features from the image. Similarly, since SIFT’s algorithm is based on analysis of high contrast points, we considered whether the darkly pigmented skin of African infants might prove more challenging. To address these considerations, we used enrollment data from participants enrolled in a longitudinal study at Chawama Clinic in Lusaka, Zambia (n=224 Zambian infants). This dataset contains all of the variables present in a pediatric clinic setting: all images were collected from infants who were under 6 months of age, in a clinic setting, over a period of about 2 months. Lastly, all of these analyses were run using the Android OS version of the SEARCH system, the goal being to test it under conditions that approximated how it would be used in routine clinical practice.

Android OS results from applying all techniques used in the MOS analysis to our Zambian Infant cohort dataset are shown in Table 6. In this case, concatenation was applied by combining the descriptors from one right and one left ear image to serve as the composite vector for each participant. Using all techniques in combination, we were again able to achieve near perfect matching rates.

**Table 6.** Top-1 and top-10 accuracies associated with applying different processing and database management techniques to the Zambian Infant dataset (Cohort 3) on Android OS.

Techniques applied (N=224)	Top-1	Top-10
**Manual Crop + Resize [150]**	86.16%	92.86%
**Manual Crop + Resize [150] + HE**	98.66%	98.66%
**Manual Crop + Resize [150] + Gender Filter**	89.29%	93.75%
**Manual Crop + Resize [150] + HE + Gender Filter**	98.66%	99.55%
**Manual Crop + Resize [150] + HE + Concatenation + Gender Filter**	100%	100%

## Discussion

Using three cross-sectional datasets (with multiple images taken from each participant to serve as the training and validation images), we demonstrate that our SEARCH system (Donut and SIFT algorithm with enhancements) is capable of achieving identification accuracies up to 100% (top-1 and top-10). Even under variable and challenging imaging conditions, the aforementioned experiments suggest that a relatively simple, yet robust method of ear identification can be leveraged as a reliable mHealth tool for patient identification on a smartphone meeting minimal requirements (have a rear-facing camera and use Android OS version 5.0 or higher). In Zambia, 96% of mobile phones meet these requirements ^19^.

In particular, as shown in Table 4 with a cohort of 224 Zambian infants in a Zambian clinic setting, we achieve 100% top-1 and top-10 performance rankings. This high predictive estimate for correct identification in a Zambian clinic setting signals that the optimized SEARCH system is a viable method for patient identification. Since mobile platforms are also used for electronic medical records, integration of the Android-based SEARCH system with a given EMR could significantly improve the utility of the latter by answering that all-important first question: who is this person?

In addition, (as shown with the MOS dataset) it is reasonable to expect that even with mechanical optimization of the Donut, image quality will degrade under real-world conditions, where there are many users (with different degrees of training) collecting data over longer periods of time. We found, however, that our ear identification algorithm can be optimized to deal with these variables. Using the MOS dataset and our Python algorithm, we tested and implemented a number of pre- processing, post-processing, and database management techniques. Each of these techniques were also implemented on our Android deployable application which was used to test the Zambian Infant cohort dataset. In each case, the combination of cropping, resizing, histogram equalization, concatenation and the application of a gender filter drastically and consistently improved identification accuracies. Near perfect identification rates were achieved in settings of non-ideal imaging conditions, and further in a dataset representing our use-case population.

Centralizing patient records hinges on the ability to correctly identify patients. Particularly, in the under-five Zambian infant population, more traditional methods of identification (such as names, birthdates, and national registration numbers) either don’t exist or have been proven unreliable. The current method for record management skirts the issue of unreliable identification methods, instead placing the burden on the caregiver to keep and maintain a clinic-issued “under-five card”. Nationwide stock-outs of the cards are also a frequent occurrence in clinics throughout Zambia ^20^. The one advantage of the under-five card is that it removes ambiguity about who the infant is.

The critical weakness, however, is that these cards are easily lost or degraded, and there is no back up for the information they contain, making loss of data irretrievable. These considerations are the primary motivator in Zambia for migrating to a centralized, clinic-based EMR. We show that ear biometrics could add significant value if implemented as a patient identification tool to link patients to a centrally based record management system.

Beyond the specific use case of replacing the current system of decentralized record management using under-five cards with a centralized, clinic-based EMR, we can envision other situations in which biometric identification would potentially be very helpful. For example, SEARCH could be very useful for cohort management in clinical research projects. In our own case, participant identification currently relies on study-issued ID cards or stickers on the under- five card, and these have the same vulnerabilities as in routine patient care. Since the SEARCH data can be aggregated at multiple levels, it could also be used to assist in tracking and identification of displaced or refugee populations, for tracking individuals after a natural disaster, for linking mobile clinic care to a central system, or as a tool to help combat human trafficking.

Careful consideration of the ethics of identification are paramount, since there are obviously ways that such technology could be misused and violate human rights. However, this is not a unique concern, but applies to all forms of biometric identification.

It may be the case that reliability of ear identification will degrade when dealing with longitudinal data – factoring in infant ear growth. A longitudinal Zambian infant cohort study launched by Project SEARCH has the specific aim of assessing how ear growth affects identification rates in infants from 0-9 months. Data collection from this study is complete and analysis is on-going. Because we have proven from the above experiments that identification rates in cross-sectional data are reliably high, we can attribute a decrease in performance to ear growth. In addition to studying the effect of ear growth on identification, there is a need to test the SEARCH system in a large-scale study when integrated with an EMR, which is currently one of our goals in the next round of field work. Further refinements to the SEARCH system will also be required, including the need to replace our current system of manually cropping images with an automated crop.

Currently SEARCH takes about a minute from image capture to the generation of a ranked list of matches, and further refinements to the system will be needed to decrease processing time.

Lastly, it would be helpful to learn more about the value of SEARCH by studying it under actual use in clinical or research settings.

## Methods

This section describes the creation of the three datasets, the SIFT pattern recognition algorithm, the matching pathway, and an explanation of computer science techniques implemented to optimize our algorithm.

### Donut design overview

Design specifications of the original donut (v1) are thoroughly detailed and design decisions justified in a previous publication ^15^. Throughout this study, design modifications were made to the original donut. These changes are outlined in Figure 1. 

### Dataset acquisition

**Boston University dataset (Cohort 1)^21^**

*Study duration:* Data collection lasted one month. Start date: 03/2017, End date: 04/2017

*Study setting:* Boston University - Charles River Campus

*Study participants:* 194 individuals: students and professors at the Boston University College of Engineering. Participants were recruited before and after the senior design classes held in the Biomedical Engineering and Mechanical Engineering Departments.

*Inclusion/exclusion criteria:* Participants were required to be over 18 years of age and willing to participate in the study.

*Ethics:* Because all data was de-identified at the point of collection, a waiver of written consent from the Boston University Institutional Review Board (IRB) was obtained. Approval/Reference number: H-35788

*Donut specifications:* Photos were taken using the first Donut (v1). The phone’s camera placement was fixed using a case permanently adhered to the Donut, the planar angle of the image was kept constant using a bubble-level, and the illumination of the photo was kept constant by using a combination of spray paint on the exterior of the Donut (to block out ambient light) and an LED strip, powered by a 9V battery, laid along the inner circumference of the Donut.

*Data collection:* All images were taken by a single, well-trained data collector. From each participant we collected two images of the left ear in a vertical position. Additionally, we collected demographic information for each participant using an excel spreadsheet. Information was de-identified by assigning a participant ID to everyone in the study. Demographic information collected included race/ethnicity, gender, and age.

*Challenges:* Hair occlusion was present in many images. Additionally, the Donut handle was not ergonomic, and difficult to hold at times.

**Museum of Science dataset (Cohort 2)^21^**

*Study duration:* Data collection spanned a period of 12 months. Start Date: 11/2018 , End Date: 11/2019

*Study setting:* Museum of Science in Boston, MA USA.

*Study participants:* 238 individuals: visitors to the Living Laboratory at the Museum of Science.

*Inclusion/exclusion Criteria:* Participants were required to be over 4 years old. Anyone under 18 years old, was required to have a parent/guardian present to sign the IRB-approved permission form.

*Ethics:* Written parent permission and consent forms were approved by the Boston University IRB and the Museum of Science, Living Laboratory team for data collection. Approval/Reference Number: H-35788

*Donut specifications:* Photos were taken using an updated version of the Donut (v2). The Donut design was updated to include a shroud which helped to standardize the location of the ear in the photographs. The shroud also helped to limit the number of extraneous features in the photo such as skin and hair.

*Data collection:* Images were taken by six different data collectors. From each participant we collected four images of the left ear, and demographic information, including age, gender, and race/ethnicity. We developed a simple data collection form using CommCare (extended data ^21^). 

*Challenges:* Demographically heterogenous cohort. The six data collectors had varying degrees of training on how to properly shroud the ear when taking an image. Battery dimming resulted in variable image quality.

**Zambian Infant dataset (Cohort 3)^21^**

*Study duration:* Data collection spanned a period of about 2 months. Start Date: 11/2019, End Date: 01/2020.

*Study setting:* Chawama Clinic in Lusaka, Zambia.

*Study participants:* 224 infants, attending Chawama Clinic for either a 6-Day vaccination visit, or the 14-week vaccination visit. 

*Inclusion/exclusion criteria:* All participants had to be attending Chawama Clinic for a vaccination visit, and planning to attend well-child visits at Chawama in the future.

*Ethics:* Written consent forms were approved to collect non-medically sensitive data from participants in this study. All forms were translated into two local languages, and approved by both the BU IRB and University of Zambia Board of Ethics. Approval/Reference Number: H-38650

*Donut specifications:* Photos were taken using a sized-down version of the Donut, termed the ‘Munchkin’ (v3), to accommodate infant ears. The distance from the camera to the ear was maintained for camera focus length. The circumference of the Munchkin is roughly half that of the Donut. The handle was also redesigned to point outward in the direction of the nose to avoid hitting the infants’ shoulders.

*Data collection:* Images were taken by one data collector, who was thoroughly trained and replaced the 9V battery on a bi-weekly basis. Two images of the left ear, two images of the right ear, and demographic information including age, gender, and weight were collected from each participant. We developed a separate data collection form using CommCare for this cohort (extended data ^21^).

*Challenges:* The clinic environment posed its own unique challenges. Images were captured while participants were in transition between getting vaccinated and being weighed. Images had to be captured in a timely manner. Since infants were coming directly from being vaccinated, they could be irritable, which at times resulted in off-centered or blurry images. Additionally, this dataset was the first where, throughout, ears were small and skin pigmentation was dark.

### Image analysis – Scale Invariant Feature Transformation (SIFT)

Former work under Project SEARCH established proof of concept that an image of the ear could be used as a biometric identifier. In these early-stage experiments, the SEARCH team tested a number of algorithms using ear images taken from the IIT Delhi database. These early experiments found that Scale Invariant Feature Transform (SIFT) was a good candidate for performing pattern recognition algorithm for our use-case ^16, 20, 22^.

Given an image, SIFT will first detect local regions of interest called “key points”. Key points are used to represent the object(s) in a given image, in our case, the ear. Next, key points are converted into a vector of real values, called “descriptors”. Each vector can be compared to other vectors (representing other images) by computing the Nearest Neighbor using squared Euclidean distance as the distance metric. An average distance between individual descriptors in a vector map is computed. A small average distance is indicative of a strong match, while a larger distance between vector maps represents a weaker match.

**Implementation**

For all validation experiments, SIFT was implemented on two software platforms: Python (an open-source, experimental platform) and Android OS (the functioning application). Our Python implementation is easily modified, and provides an efficient way to test any changes made to the algorithm. Experiments on Python are run using a computer, where Python is installed, and datasets of ear images are saved to a local folder. 

Our Android implementation is built with the intention of benchmarking the performance of techniques tested on the Python platform. This application uses the same library, OpenCV, as the Python platform and all the same algorithms for the sake of parity. This application takes in training and testing datasets and attempts to match each image in the testing set against the training set. The rank-1 to rank-10 recognition rates were recorded. The BU dataset was the first to be tested, followed by the Museum of Science Dataset and the Zambian Infant Cohort Enrollment Dataset.

**Operation**

In order to run tests using our Python implementation, Python (version 3.6.9) is required, and folders of testing and training data must be labeled and their directories specified.

In order to run our Android implementation, Android OS version 5.0 or higher is required. The version of OpenCV used is 2.4.11. The training and testing images are loaded in from a local directory one at a time. The images are put through the detection pathway, resulting in a set of image descriptors for each image. To save time in the event that a test is rerun, these descriptors are stored within a local SQLite database. On subsequent test runs with the same dataset, the feature extraction step is skipped entirely. Matching then follows and lastly the rank-1 and rank-10 recognition rates are then computed and written to a csv file.

### Matching pathway

**Figure 3.**
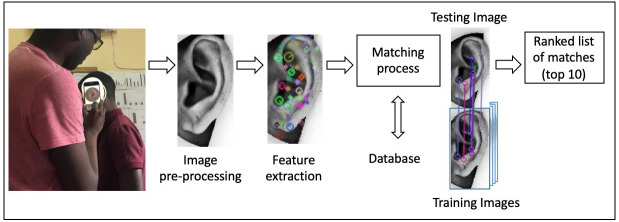
Matching pathway for the SEARCH system. Moving from left to right, we start by taking an image using a smart phone mounted on the back of the image stabilizing Donut. The Donut serves to reduce random variation during the image capture step, and does so by standardizing distance, angle and lighting intensity, and by eliminating vibration of the phone relative to the ear. Once the image is captured it is converted automatically to gray scale for SIFT analysis. SIFT extracts a series of descriptors, high contrast features on the image, and uses these to construct a vector map of the ear. This vector map is what is used subsequently to seek matches within the data base. If this is the first time entering the system, the vector map is entered as a new individual. If this is a return visit, the database can be queried to identify the most likely matches based on comparison of Euclidean distances from the test image to the database of stored vector diagrams. The results are then displayed as a top-10 ranked list of the most likely matches. At this point, the user selects the correct match and proceeds with data entry in the electronic medical record.

Here, we describe the matching pathway that was taken for each dataset, depicted in Figure 3. First, data are collected using the Donut. At least one training and one testing image are designated from these data. The training image(s) is then converted into a vector map (containing descriptors for points of high contrast on the image) and stored in a database. What this means is that the SEARCH system does not actually store pictures of the ears, just the vector maps.

This reduces data storage requirements to a significant degree, and also removes the possibility that someone who gained access to a phone running SEARCH could somehow identify individuals by inspecting the photographs of their ears. The testing images are also converted into vector maps of descriptors. For each participant, testing data is matched to a vector stored in the training database. Average Euclidean distance values between the testing vector and each vector stored in the training database are computed. The smallest distances correspond to the strongest matches. Finally, a ranked list of the top 10 strongest matches (10 smallest distances) are displayed. If the correct match is listed as the strongest (first) match, it is designated as a top-1 and top-10 match. If the correct match is contained within the top-10 list, but is not first on the list, this is designated as a top-10 match. If the correct match is not contained within the top- 10 list, it is designated as no match.

### Improvements to the detection and matching pathway

Here we describe the computer science techniques applied to the SEARCH algorithm to deal with variables present in our datasets.

**Manual crop**

A manual crop was applied to all images prior to running them through the SEARCH algorithm. This was done using the image processing toolbox in MATLAB, and saving the newly cropped images into a new folder, which was then fed into the SEARCH algorithm. A manual crop isolates the ear, reducing background noise in the image and increasing the proportion of the image that contained the ear. This ensures that SIFT features come from the ear, and not surrounding background information.

**Resize to smaller pixel count**

A resize was applied after cropping images in all three datasets. Images were resized as part of the pre-processing techniques within the SEARCH algorithm. In our case, manually cropped images were resized to a set width of 150 pixels and a height that maintained proportions of the original (cropped) images. Dimensions of the full-size cropped images had an average of ~2000 × ~1000 pixels, which were resized to a set 150 pixels × ~75 pixels (dependent on the initial size ratio of the cropped image). This ten-fold decrease in size helped to improve processing speed of the algorithm dramatically – reducing the time to process 238 images from ~40 minutes to <1 minute. Additionally, resizing has a similar effect to that of a median filter or Gaussian blur, commonly applied in image detection. Resizing to a much smaller pixel count limits noise in the image, constraining SIFT point detection to the most distinct features. When coupled with a manual crop, this results in a dramatic increase in the SEARCH algorithm’s performance.

**Histogram equalization**

Literature points out that histogram equalization is helpful with SIFT-point-based matching algorithms, by improving the number of matching key points between two images ^23^.

**Figure 4.**
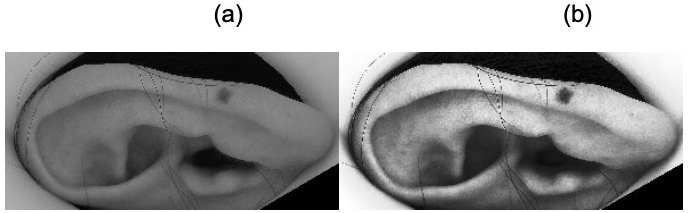
Example of the application of histogram equalization to an image of an ear. The image on the left is before histogram equalization. The image in the right is after applying histogram equalization. The net effect is a significant intensification of contrast in the latter image. Edges are much clearer. This is helpful to SIFT which works by identifying regions of interest by contrasting the intensity of adjacent pixels in an image. This typically has the effect of making SIFT sensitive to the detection of borders or the edges of objects, such as the margin of an ear, or the contours of the inner cartilage.

Histogram equalization is a common image enhancement technique that has been seen as part of the pipeline of 2-D ear recognition ^23^. The main purpose is to lower illumination-induced variability of different ear images. This pre-processing technique is also beneficial to our system. Conceptually, histogram equalization is a three-step technique. First, we compute the intensity histogram of the given binary image. Then, we spread out the most frequent intensity values to the less frequent intensity values, thus making an "equalized" histogram. Lastly, we change the intensity value of every pixel, from its corresponding intensity in the old histogram to the equalized intensity in the computed histogram ^24^. By applying histogram equalization, we can increase the contrast of the low contrast areas in a greyscale image. The image will be less blurry, and the boundaries of objects in the image will become more distinguishable ( Figure 4).

**Key points concatenation**

Key points are features detected by the SIFT algorithm used to represent the identity of ear images. They are later converted to a vector of 128 numbers (descriptors),that allows the computation of their distance score. Distance score is the squared loss of the two descriptors to be matched, and it describes how “different” two descriptors are. In other words, distance scores tell us how likely the two ear images are the same ear.

In an ideal condition, images of different ears should have high distance scores while images taken from the same ear hold a low distance score. During this process, features that are unique to a particular ear make a distance score between the ear and any other ears high. As an opposite result, features that are common among different ears only contribute little to the distance score between images of different ears. SIFT finds both unique and common features. Therefore, we need to find as many unique features from an ear as possible in order to increase the distance score between different ear images. One solution to doing this is through concatenating descriptors converted from the key points of the same ear ^24^. After that, we match these combined descriptors with other concatenated descriptors created in the same way. Since we collect at least two images from different angles of a participant, we are able to combine key points detected by the two images to calculate the descriptors generated by these key points. That is to say, we are able to use unique key points from both images for ear matching, instead of from only one image.

**Gender filter**

At the time of image capture, we also record the gender of the participant. This information allows us to separate participant data into gender-specific groups. Matching is then performed between people in the same gender group, reducing the size of the searchable database. By narrowing the database based on gender, we significantly reduce the total number of possible matches, decreasing the chances of a false-positive and increasing the probability of a correct match.

### Statistical analysis

The nature of this study is that of an iterative benchmarking analysis. With each cross-sectional dataset, we tested the performance of our algorithm and made changes to the matching pathway as detailed above. Top-1 and top-10 matching performance rates were determined by summing the total correct matches and dividing this number by the total possible matches for each dataset. There was no statistical precision, due to the self-contained nature of each dataset.

### Open alternatives

Throughout this study, all images were cropped using the proprietary software: MATLAB. This could be done using any open-source photo editing software, such as Inkscape or Gimp. Our Python and Android OS algorithms can be found in the repository specified in the software availability section ^25^. These both operate using open source software.

## Consent

Written informed consent for publication of the participants’ details and/or their images was obtained from the participants/parents/guardian/relative of the participant.

## Data availability

### Underlying data

Zenodo: Ear Datasets - BU, MOS, ZIC. http://doi.org/10.5281/zenodo.4147637 ^21^

This project contains the following underlying data:

- BU Dataset (n=194 participants from Boston University, 2 images of each participant’s left ear, cropped)
- MOS Dataset (n=238 participants from the Boston Museum of Science, 4 images of each participant’s left ear, cropped)
- Zambian Infant Cohort Dataset (n=224 participants from the Chawama Clinic in Zambia, a total of 4 images of each participant’s left and right ears)

### Extended data

Zenodo: Ear Datasets - BU, MOS, ZIC. http://doi.org/10.5281/zenodo.4147637 ^21^

- Extended Data (CRFs for data collection at the MOS and Zambian Infant Cohort Study).

### Reporting guidelines

STROBE checklist for ‘Project SEARCH (Scanning EARs for Child Health): validating an ear biometric tool for patient identification in Zambia’ http://doi.org/10.5281/zenodo.4068738 ^26^

Data are available under the terms of the Creative Commons Attribution 4.0 International Public License.

## Software availability

Python code and the Android application are only available from Zenodo

Archived source code at time of publication: http://doi.org/10.5281/zenodo.4091658 ^25^

License: Creative Commons Attribution 4.0 International
