# Supplementary material for: Project SEARCH (Scanning EARs for Child Health): validating an ear biometric tool for patient identification in Zambia
Source: Gates Open Res. 2020 Nov 6;4:168. [Version 1] doi: 10.12688/gatesopenres.13197.1 (PMC7887869; doi:10.12688/gatesopenres.13197.1)
Supplement: Peer review history

# Peer review: Reviewer response for version 1

**DOI:** 10.21956/gatesopenres.14405.r30337

**Published:** 2021-02-16

**Authors:** Nino L. Wouters

**Affiliations:** 1 Department of Clinical Technology, Faculty of Mechanical, Maritime and Materials Engineering (3Me), Delft University of Technology, Delft, The Netherlands

Very relevant article, congratulations! It was a very interesting and concise read. The problem of identification is a renowned problem in field work in developing or rural countries. Therefore, a reliable, low cost and foolproof method is an essential primary concern. This paper is therefore a valuable contribution for future research and studies performed in LMICs. Your introduction underlines this valuable contribution very well. However, I would suggest to cite and describe more attempts or current methods addressing the problem of identification in order to anchor your article more securely to the current state of affairs. Make the reader feel the urgency for such an identification method. I was very interested by the technical details of your design process and very pleased to read the detailed description of the problems that led to each iteration. The same goes for the method of testing your system: an accurate description of the population paired with the analysis led to a convincing scientific result. I would be interested to read any future prospects you have with this system. Has it already been used as an identification method in recent studies? Have you thought of a way for storing the data should it be commonly used?

 Thank you again for this interesting and relevant contribution.

Is the work clearly and accurately presented and does it cite the current literature?

Partly

If applicable, is the statistical analysis and its interpretation appropriate?

Yes

Are all the source data underlying the results available to ensure full reproducibility?

Yes

Is the study design appropriate and is the work technically sound?

Yes

Are the conclusions drawn adequately supported by the results?

Yes

Are sufficient details of methods and analysis provided to allow replication by others?

Yes

Reviewer Expertise:

Cross-sectional studies, identification techniques, LIMCs, Audiology

I confirm that I have read this submission and believe that I have an appropriate level of expertise to confirm that it is of an acceptable scientific standard.

# Peer review: Reviewer response for version 1

**DOI:** 10.21956/gatesopenres.14405.r30237

**Published:** 2021-01-26

**Authors:** David S. Carrell

**Affiliations:** 1 Kaiser Permanente Washington Health Research Institute, Seattle, WA, USA

This study addresses a compelling issue regarding accurate identification of patients in low- and middle-income countries: the need for a biometric identification technology that is robust, relatively simple, accurate, and easily implemented in populations of infants.

 The writing is very clear and presented in a narrative style that allows the reader to appreciate the challenges that were encountered in the course of this work and how the study team responded to those challenges. This style of writing is a strength of the paper. For example, the impact on image quality of weak 9-volt batteries and how this led to future adaptations in image processing was interesting.

 The findings presented in this paper are very promising and support continued development of this technology and its real-world evaluation. Future work should consider performance of the technology using larger patient samples wherein the possibility for apparent but false-positive matches would be increased. Because patient sex is used to bifurcate the set of potential matches, the effective sample sizes in the Zambian data sets is 109 girls and 116 boys. These are relatively small sample sizes compared to the size of patient cohorts in clinics where such technology would be used for identification.  

 The question as to how this technology would perform longitudinally, as subjects age and ear topography potentially changes, is also important, and as the authors report is currently being investigated in another data set.

 The authors should consider addressing the following minor comments in a revised version of this paper:

1. A definition of “top-1” and “top-10” should be provided at first use of these terms; it is not until the methods section that their definitions become clear.
2. The real-world value to a healthcare practitioner of a top-10 list of potential matches to a given patient is not adequately described. How such a list would be used in practice to identify the correct patient record should be described (even if illustrated with hypothetical data to preserve patient privacy).
3. Some readers of this work may be curious to know if this technology has any possibility of success without the use of a Donut. Clearly, the Donut is important, as described. But the authors’ opinions about a potential future technology that would not require the Donut would be of interest, as the necessity of the Donut clearly limits the technology’s utility (though not in a fatal way).
4. The caption for Figure 2 appears to include, at the very end, some residue of template text that should be deleted.
5. Including Table 2 is fine, but it may not be strictly necessary given it reports only two data values and both are presented in the narrative.
6. Reporting results without considering the sex of the patient in the Zambian data would provide some insight into performance of the technology in a larger pool of patients (approximately double in size). This would simulate results in a larger data sets without having to collect any additional data.
7. An explanation is needed as to why the use of both ears (one left and one right) was introduced in the Zambian data. What led to this decision and why might it be superior to using two images from one ear?
8. In a real-world application would the photos themselves be stored centrally, or just the vector representations, and what are the potential privacy issues, if any, of storing the actual photos? Might there be value in terms of confirming identification of storing the actual photos (e.g., if a human compared the photo to the patient)?
9. How does ear-ID degrade over time as a person matures, especially a very young person?
10. Are there potential privacy issues in using a ranked set of 10 potential matches?
11. Figure 3 caption: What are the implications, if any, of a healthcare practitioner not having knowledge as to whether the immediate visit is the patients 1st visit?
12. Methods, “Open alternatives” section: While it is likely that cropping with an open source tool will yield similar results, this should be attempted and confirmed.

Is the work clearly and accurately presented and does it cite the current literature?

Yes

If applicable, is the statistical analysis and its interpretation appropriate?

Not applicable

Are all the source data underlying the results available to ensure full reproducibility?

Yes

Is the study design appropriate and is the work technically sound?

Yes

Are the conclusions drawn adequately supported by the results?

Partly

Are sufficient details of methods and analysis provided to allow replication by others?

Yes

Reviewer Expertise:

In my research I use clinical natural language processing technologies and machine learning methods to develop models to predict patient phenotypes (e.g., whether the person has experienced anaphylaxis, a substance use disorder, or has some clinical condition such as major depression, diabetes, or carotid artery stenosis.  These investigations are heavily dependent on secondary use of actual electronic health record data from large cohorts of patients.

I confirm that I have read this submission and believe that I have an appropriate level of expertise to confirm that it is of an acceptable scientific standard.
